# Modifiable factors associated with bone health in Malaysian adolescents utilising calcaneus quantitative ultrasound

**DOI:** 10.1371/journal.pone.0202321

**Published:** 2018-08-14

**Authors:** Mohamed S. Zulfarina, Razinah Sharif, Syed-Badrul Syarifah-Noratiqah, Ahmad M. Sharkawi, Zaris-SM Aqilah-SM, Sabarul-Afian Mokhtar, Shuid A. Nazrun, Isa Naina-Mohamed, on behalf of the MALBONES research group

**Affiliations:** 1 Pharmacoepidemiology and Drug Safety Unit, Department of Pharmacology, Faculty of Medicine, UKM Medical Centre, Universiti Kebangsaan Malaysia, Cheras, Kuala Lumpur, Malaysia; 2 Nutritional Sciences Programme, Faculty of Health Sciences, Universiti Kebangsaan Malaysia, Kuala Lumpur, Malaysia; 3 Department of Orthopaedic, Faculty of Medicine, UKM Medical Centre, Universiti Kebangsaan Malaysia, Cheras, Kuala Lumpur, Malaysia; Augusta University, UNITED STATES

## Abstract

Maximizing bone mineral accrual to attain an optimal peak bone mass (PBM), particularly during adolescence, appears to be an effective protective strategy in the prevention of osteoporosis. This study aimed to evaluate the influence of physical activity (PA), fat mass (FM), lean mass (LM), body mass index (BMI), calcium, or combination of vitamin D supplement intake, smoking and alcohol drinking status on bone health assessed by calcaneus quantitative ultrasound (QUS) in a healthy adolescent population. The participants comprised of 920 male and female secondary school adolescents aged 15–17 years old. Quantitative ultrasound measurements of the left heel were performed using Lunar Achilles EX II, which included results of broadband ultrasound attenuation (BUA), speed of sound (SOS), and a calculated stiffness index (SI). Multivariable linear regression analyses revealed that—PA was positively associated with all three QUS indices in both genders; BMI was positively associated with SI and SOS in females; LM was positively associated with BUA in both genders; and FM was negatively associated with SI in females. These variables accounted for 32.1%, 21.2% and 29.4% of females’ SOS, BUA and SI variances (p<0.001), respectively and 23.6%, 15.4% and 17.2% of males’ SOS, BUA and SI variances (p<0.001), respectively. Promoting health benefits from physical activity could influence bone status and consequently improve PBM, which is a potent protective determinant against osteoporosis in adulthood.

## Introduction

As we age, bone mass reflects the difference between the level of peak bone mass (PBM) attained during growth and the rate of subsequent age-related or postmenopausal bone loss. Depending on the skeletal site, the peak bone mass is estimated to be achieved by the end of the second or early in the third decade of life [1]. Once PBM has been attained, ensuing bone gains are minimal and bone mass gradually diminishes with age.

There is considerable evidence that the acquisition of bone mineral during growth is critical in determining bone mass in adulthood [2]. It has been proposed that maximizing bone mineral accrual during adolescence to attain an optimal PBM appears to be an effective protective strategy in the prevention of osteoporotic fractures in later life [3–5]. Adolescence is a particularly important period to maximize bone accrual given that the skeleton undergoes rapid changes owing to the continuous formation of bone that exceeds bone resorption, a process known as bone modelling [1]. During the 2 years of peak skeletal growth, adolescents accumulate over 25% of their future PBM; and by the age of 18 years in females and 20 years in males (late adolescence), up to 90% of adult PBM has been acquired [6,7]. As only the remaining 10% of bone consolidation occurs later, adolescence can be seen as a critical time for rapid skeletal growth and attainment of PBM.

By maximizing PBM, the risk of developing osteoporosis-related fracture may be reduced by 50%, whereby a 10% increase in PBM corresponds to an increase of one standard deviation in bone mineral density (BMD) [2, 8, 9]. Based on computer simulations of bone loss over life, the onset of osteoporosis is extrapolated to be deferred by thirteen years if the BMD of a young adult is 10% higher than the mean [10]. Although 60–70% of the PBM variance is mostly regulated by genetic factors [11], studies have shown that environmental factors such as nutritional intake of calcium and/or dairy products as well as physical activity (PA) [12, 13] may play a vital role in modulating and enhancing adolescent bone mass accretion beyond the genetic potential of their bones [14, 15]. Low body mass index (BMI) is a known modifiable risk factor for osteoporotic fractures [16]. However, findings regarding the contribution of body composition components, which include fat mass (FM) and fat free mass or lean mass (LM), on bone health measures using QUS were inconclusive and warrants more research.

Because adult bone mass depends largely on the PBM attained during earlier life, the aptitude to identify adolescents who could be at an increased risk as a result of low bone accretion is another important strategy to curb the occurrence of osteoporosis and fragility fractures in later stages of life. Based on the definition of osteoporosis by the World Health Organization (WHO) in 1994 [17], BMD assessed by dual energy x-ray absorptiometry (DXA) is currently recognized as the reference or gold standard in the diagnosis of osteoporosis [18]. However, DXA measurement is 2-dimensional, which varies with bone size and unable to measure bone microarchitecture that contributes to the overall bone strength and fracture risk [19–22]. On the other hand, quantitative computed tomography (QCT) can be used to assess the actual volumetric BMD. However, QCT involves exposure to unnecessary ionizing radiation that limits its routine application and its use in large population studies [19–22].

In an ideal context, there should be a safe and practical method to assess bone health status, particularly in a large community-based study. Quantitative ultrasound (QUS) is gaining popularity because it is convenient, fast, cost-effective, portable and radiation-free, if compared to DXA and QCT. Calcaneus QUS measurements have been demonstrated to correlate highly with BMD measured by DXA in elderly men and women [23], in healthy paediatric populations [24], as well as other properties of bone including bone microstructure, bone elasticity and bone connectivity [25, 26]. These measurements are thought to depend not only on bone mineral density but also on spatial orientation of the bone trabeculae which contributes to the mechanical strength of bone that is pivotal in the evaluation of fracture risk [27].

Therefore, the identification of adolescents at risk of low bone accrual as well as modifiable factors (which can maximise PBM during adolescence) that contribute to QUS measurements are of paramount importance in the prevention of osteoporosis and risk of fragility fracture. However, in contrast to the abundance of DXA-related studies, studies pertaining to the influence of modifiable factors of adolescents’ bone health measured by QUS of the calcaneus were limited. To our knowledge, no study has been reported about the factors influencing bone gain using Achilles QUS among Malaysian adolescents. Therefore, the purpose of this study was to determine the influence of BMI anthropometric, body composition (including fat mass and lean mass) and lifestyle factors (such as physical activity, smoking, alcohol drinking status and calcium, combination with vitamin D supplement intake) on bone health measured by calcaneus QUS in a large population of healthy Malaysian adolescents.

## Methodology

### Study design

The data used in the present study was from the first phase of the Malaysia Bone Health and Osteoporosis study (MALBONES) group. The MALBONES study was a population-based cross-sectional study conducted among Malaysian adolescents in the Peninsular Malaysia. Field data collection was conducted from September 2014 until July 2015.

### Study area

This study was conducted in the central and eastern regions of Peninsular Malaysia, namely the Federal Territory of Kuala Lumpur (metropolitan area), and Selangor (central region) and Pahang (eastern region) state. These two states and one metropolitan area were chosen based on discussions with the Ministry of Education of Malaysia.

### Study population

The study population was representative of the adolescents in Peninsular Malaysia. The participants of this study comprised of both male and female students aged 15–17 years old, who were in their fourth year (form four) in public secondary schools.

### Sampling

This study employed a stratified random sampling design. First, an updated list of the government secondary schools situated in the selected regions was acquired via the Ministry of Education of Malaysia’s official portal [28]. The list was stratified into the different types of school system as classified by the Ministry of Education of Malaysia [28] and was used as the sampling frame. The sampling frame consisted of four groups. The first group was daily school (both urban and rural area) and vernacular schools. The second group was full-boarding school. The third group was sports school and the final group was half-boarding school (a mixed school system comprised of both daily and boarding school systems) such as the technical and vocational schools. In summary, data collection was carried out in 6 public secondary schools, whereby each of the different types of schools (daily school-urban, daily school-rural, boarding, daily-vernacular, sports school and Orang Asli dominated school) was randomly selected using computer-generated random number lists to represent the different niches based on the national school system, location and ethnicity.

All students in their fourth year in the selected secondary schools were invited to participate in this study. As a requirement, students must be able to read and write in the national language of Malaysia. The exclusion criteria were students with any type of chronic diseases or metabolic bone disease or who were taking medications or treatment that could affect growth or bone metabolism and as well as those who presented with any apparent signs of mobility impairment. All participation was voluntary, although only students who attended schools during the data collection day and submitted the agreed written consent forms (participants and their parents or guardians) were recruited into the study.

### Ethics statement

All participants and their legal guardians were presented with information sheets detailing the study. Written informed consent was obtained prior to participation, which indicated their willingness to participate. The protocol for this study was designed according to the guidelines established by the Declaration of Helsinki and ethical approval was obtained from the Medical Ethics Committee of the University Kebangsaan Malaysia Medical Centre, Malaysia (UKM 1.5.3.5/244).

### Measurements

#### Demographic profile

The participants’ sociodemographic information such as age, gender, date of birth, ethnic group and type of school were collected via questionnaire.

#### Fracture history

Fracture history during the preceding year was assessed. If there was history of past fracture, the participant was asked to give details about the part of bone involved.

#### Supplement intake

Intake of vitamin or mineral supplementation in the previous 12 months was evaluated. Specific intake of calcium, or combination with vitamin D was extracted according to Anatomical Therapeutic Chemical (ATC) code A12A, a standard for drug utilization research developed by the WHO Collaborating Centre for Drug Statistics Methodology [29].

#### Tobacco smoking

Based on the definition by the National Health and Morbidity Survey (NHMS), current smoker (smoked any tobacco products either daily or occasionally at the time of the survey) was defined as respondents who smoked for the past 12 months [30]. Current smoker who smoked sometimes (i.e. not every day) was categorized as occasional or social smoker, whereas current smoker who smoked at least one cigarette per day (cpd) was categorized as daily or regular smoker. In the current study, “ever” smoker (i.e. tried it once or just one puff), never smoker (i.e. never smoked in the lifetime) and former smoker (i.e. used to smoked in the past but had quit smoking for the previous 12 months) were combined into the non-smoker category.

#### Alcohol consumption

In accordance with NHMS 2011, current drinker was defined as a respondent who had consumed any alcoholic beverages for the past 12 months [30]. Current drinker who drank once in a while or less than once in a month was classified as occasional or social drinker, whereas current drinker who drank at least once in a month or more frequently was classified as regular drinker. For the current analysis, “ever” drinker (i.e. tried it once or twice), never drinker (i.e. never tried drinking in the lifetime) and former drinker (i.e. consumed alcohol in the past but had stopped drinking for the previous 12 months) were combined into the non-drinker category.

#### Physical activity

Adolescent physical activity level was evaluated using the short-form International Physical Activity Questionnaire (IPAQ) in Malay language [31]. The IPAQ has been validated for use in age-range of 15–69 year and was found to have high reproducibility [32]. In brief, IPAQ estimated the overall physical activity level by assessing the frequency (numbers of days per week) and duration (minutes per day) in which subjects spent more than 10 minutes performing different types/levels of intensity (walking, moderate-intensity activities and vigorous-intensity activities) of physical activity undertaken during the previous 7 days. The level of physical activity was computed in terms of multiples resting metabolic rate or metabolic equivalent (MET)-minutes (METs) per week (MET-minutes/week). The MET levels were 8.0, 4.0, and 3.3 for vigorous intensity activities, moderate intensity activities, and walking activities, respectively. The total or overall physical activity score was the summation of all METs per week (MET-minutes/week) from walking to moderate-intensity activities to vigorous-intensity activities.

#### Anthropometric and body composition

Height was measured in triplicate using a calibrated portable Stadiometer model 213 (Seca, Germany) to the nearest 0.1cm without shoes and socks. The body weight was measured using a portable body composition analyser SC-330 (Tanita Corporation, Japan) to the nearest 0.1 kg with light clothing. Body composition measures, including lean mass (LM) and fat mass (FM), were determined using the same body composition analyser (developed for age range of 5–99 years) which uses bioelectrical impedance analysis that evaluates the resistance or impedance to signals as it travels through the water that is found in fat and muscle. The bioelectrical impedance analysis is a practical method to estimate fat content in children and adolescents and the provided estimated values were highly correlated to DXA [33, 34]. Body mass index (BMI) was calculated as weight (kg) divided by square of height (m^2^). Among female participants, they were asked whether menarche had occurred and if so, their menarche age in years was recorded [35].

#### Bone health assessment

QUS measurements of the left calcaneum were performed using a portable Achilles EX II (GE Lunar, Madison, WI, USA). All calcaneus QUS measurements were performed by the same trained operator according to the standard procedure provided by the manufacturer. The instrumental quality control was performed regularly and re-calibrated by scanning the manufacturer-provided phantom, each time it was moved. Three indices or parameters were determined from the QUS measurement: stiffness index (SI), broadband ultrasound attenuation (BUA, dB/Mhz) and speed of sound (SOS, m/s).

The first outcome parameter, BUA refers to the absorption of sound waves through the bone (dB/MHz). BUA is the attenuation of ultrasound waves that travel from the transmitting transducer to the receiving transducer. BUA represents the trabecular spatial orientation in which the value increases with greater trabecular complexity [25]. The second parameter, SOS expresses bone stiffness by the ratio of the traversed distance to the transit time (m/sec). SOS is the speed the ultrasound signal transport from one transducer to the other. SOS represents the velocity of sound travelling through the skeleton and its adjacent soft tissue in which the values increase with greater trabecular elastic modulus, while assuming constant heel stiffness [25]. A new parameter, referred to as SI, is a linear combination of SOS and BUA. SI score was derived from SOS and BUA, whereby it was calculated by the software of the device according to the following equation: SI = 0.67 X BUA + 0.28 X SOS—420. Higher SI, SOS and BUA values signified better bone health.

To compute the short-term precision in the current study, 15 subjects were measured three times on the same day by the same trained operator. Each participant’s foot was repositioned between measurements. The coefficient of variation (CV) for within-days measurements was <2.0%, which was within the acceptable value provided by the manufacturer.

### Statistical analysis

Analyses were performed separately for females and males. Unpaired t-test and one-way analysis of variance (ANOVA) with Bonferroni correction were performed to compare the significant difference between means. Multiple linear regression (MLR) analysis using the forced entry method was employed to determine the best combination of modifiable predictor for each QUS variance. Separate models were created for SOS, BUA, SI and for both sexes. Multiple regression models were created by entering the independent variables that were significant correlates of SOS, BUA and SI variance as found in bivariate (i.e. simple) linear regressions.

The models included anthropometric BMI, body composition (fat mass and lean mass) and lifestyle (physical activity, calcium, or with vitamin D supplement intake, smoking status and drinking status) as modifiable variables of interest. Age, ethnicity, school residency, fracture history and years since menarche in females were evaluated and those that were significantly correlated were included as confounding variables (covariates) in the models. The main dependent variables in the models were the calcaneus QUS parameters (SOS/BUA/SI). Height and weight were not entered into the regression models to avoid multicollinearity with BMI. The final models were assessed for normality and homogeneity of variance and the plots showed that the two assumptions were met. Results were reported as: mean± standard deviation (SD) for descriptive statistical analyses; as well as adjusted unstandardized regression coefficient (*B*), its accompanying standard error (SE) and the model’s coefficient of multiple determination (R^2^) for the multiple linear regression analyses. A p-value (2-sided) of less than 0.05 was considered to be statistically significant. All descriptive statistics and regression analyses were carried out using IBM SPSS version 22 (Armonk, NY: IBM Corp.)

## Results

### Descriptive characteristic

A total of 960 participants had complete data in the study. However, forty participants were excluded from the analysis because the following reasons—not in the acceptable age-range (n = 22); non-Malaysian (n = 2); indigenous ethnic from Sabah and Sarawak (non-resident of Peninsular Malaysia) (n = 12); and took anticonvulsants or corticosteroids (n = 4). Hence, the final study sample size consisted of 920 secondary school adolescents with a balanced representation from both genders (456 females and 464 males) and a racial distribution which resembled the ethnic distribution in the general population of Malaysia (65.4% Malay, 20.7% Chinese, 9.5% Indian and 4.5% Orang Asli). The mean age was 15.46±0.57 years with no significant differences between the two groups, 15.43±0.55 and 15.49±0.58, for females and males, respectively. All female participants had reached their menarche (post-menarche). Table 1 displays the descriptive statistics of the characteristics of the study sample by gender.

**Table 1:** Participant descriptive characteristic.

Parameter		Female (n = 456)	Male (n = 464)	Total (n = 920)
		Mean±SD / N(%)	Mean±SD / N(%)	Mean±SD / N(%)
**Age (years)**		15.43±0.55	15.49±0.58	15.46±0.57
**Ethnicity**	**Malay**	311(68.2)	291(62.7)	602(65.4)
	**Chinese**	83(18.2)	107(23.1)	190(20.7)
	**Indian**	37(8.1)	50(10.8)	87(9.5)
	**Orang Asli**	25(5.5)	16(3.4)	41(4.5)
**School type**	**Sport**	42(9.2)	59(12.7)	101(10.9)
	**Boarding**	62(13.6)	72(15.5)	134(14.6)
	**Urban**	199(43.6)	190(40.9)	389(42.3)
	**Rural**	153(33.6)	143(30.9)	296(32.2)
**Height (cm)**		155.71±6.06	166.07±6.92*	160.93 ±98.31
**Weight (kg)**		53.31±12.67	59.29±14.83*	56.33±14.11
**BMI (kg/m**^**2**^**)**		21.91±4.65	21.41±4.79	21.66±4.72
**FM (kg)**		15.965±8.26*	8.84±7.82	12.37±8.79
**LM (kg)**		35.21±4.51	47.82±7.45*	41.57±8.82
**Total PA (MET-min/week)**		1553.71±1544.11	1957.35±1736.25*	1766.37±1656.42
**Calcium, or with vitamin D supplementation**	**No**	446(97.8)	454(97.8)	900(97.8)
	**Yes**	10(2.2)	10(2.2)	20(2.2)
**Smoking status**	**Non-smoker**	456(100)	398(85.8)	854(92.8)
	**Occasional**	0	18(3.9)	18(2.0)
	**Regular**	0	48(10.3)	48(5.2)
**No. of cigarettes per day (cpd)**		0	6.25±6.0	6.25±6.00
**Drinking status**	**Non-drinker**	433(95.0)	413(89.0)	813(88.4)
	**Occasional**	23(5.0)	51(11.0)	74(8.0)
	**Regular**	0	0	0
**Bone fracture**	**No**	444(97.4)	426(91.8)	870(94.6)
	**Yes**	12(2.6)	38(8.2)	50(5.4)
**Time since menarche (years)**		3.6±1.38		
**QUS measurement**	**SOS**	1579.93±36.26	1593.49±39.75*	1586.77±38.63
	**BUA**	121.83±12.42	126.72±12.82*	124.30±12.85
	**SI**	103.43±15.17	110.13±17.72*	106.98±16.87

SD, standard deviation; BMI, body mass index; FM, fat mass; LM, lean mass; PA, physical activity; QUS, quantitative ultrasound; SOS, speed of sound; BUA, broadband ultrasound; SI, stiffness index.

*statistically significant difference between adolescent females and males (p<0.05)

There was significant difference between females and males in weight, height, LM, FM, total PA, SOS variance, BUA variance and SI variance (p<0.05). As anticipated, females had significantly greater FM than males, whereas, males had significantly greater stature, weight, LM and total PA (p<0.05) than females. However, there was no significant difference between genders regarding BMI. The mean calcaneus values for the study sample were 106.98±16.87, 124.30±12.85, 1586.77±38.63 for SI, BUA and SOS, respectively. Males had significantly higher SOS, BUA and SI values than females (p<0.05).

### Modifiable determinants of bone health

The result of multivariable regression analyses between QUS values and related predictors is presented in Table 2 for females and Table 3 for males. After adjusting for significant covariates, in the SOS model, PA and BMI in females were identified as the best significant predictors (R^2^ = 0.321, p<0.001). In males, SOS model was best predicted by PA (R^2^ = 0.236, p<0.001). In the BUA model, PA and LM were identified as the best significant predictors in both females (R^2^ = 0.212, p<0.001) and males (R^2^ = 0.154, p<0.001). In the SI model, PA, BMI and FM in females were identified as the best significant predictors (R^2^ = 0.294, p<0.001). In males, PA was the only independent predictor of SI (R^2^ = 0.172, p<0.001). Overall, these contributions accounted for 32.1%, 21.2% and 29.4% of adolescent females’ SOS, BUA and SI variances, respectively; and 23.6%, 15.4% and 17.2% of adolescent males’ SOS, BUA and SI variances, respectively.

**Table 2:** Multiple regression analyses predicting SOS, BUA and SI for adolescent females.

FEMALE	SOS (ms)^a^			BUA (dB/MHZ)^b^			SI^c^		
	*B*(SE)	p-value	R^2^	*B*(SE)	p-value	R^2^	*B*(SE)	p-value	R^2^
			0.321			0.212			0.294
**Body Mass Index (kg/m**^**2**^**)**	0.847(0.310)	0.006		0.791(0.556)	0.156		1.874(0.657)	0.005	
**Lean mass (kg)**	-	-		0.896(0.224)	0.000		0.531(0.273)	0.053	
**Fat mass (kg)**	-	-		-0.318(0.352)	0.367		-0.860(0.416)	0.040	
**Total physical activity (MET-min/week)**	0.008(0.002)	0.001		0.002(0.001)	0.032		0.003(0.001)	0.002	
**Calcium/vitamin D user (yes/no)**	-	-		-	-		-	-	
**Current smoker (yes/no)**	-	-		-	-		-	-	
**Current drinker (yes/no)**	-	-		-	-		-	-	

*B* = Adjusted unstandardized regression coefficient. SE = Standard error for the adjusted unstandardized regression coefficient. R^2^ = Coefficient of multiple determination.

^a^ Model adjusted for age, ethnicity and school residency

^b^ Model adjusted for school residency and year since menarche

^c^ Model adjusted for ethnicity, school residency and year since menarche

**Table 3:** Multiple regression analyses predicting SOS, BUA and SI for adolescent males.

MALE	SOS (ms)^a^			BUA (dB/MHZ)^b^			SI^c^		
	*B*(SE)	p-value	R^2^	*B*(SE)	p-value	R^2^	*B*(SE)	p-value	R^2^
			0.236			0.154			0.172
**Body mass index (kg/m**^**2**^**)**				0.038(0.500)	0.538				
**Lean mass (kg)**				0.547(0.137)	0.000				
**Fat mass (kg)**	-0.395(0.215)	0.066		-0.373(0.284)	0.190				
**Total physical activity (MET-min/week)**	0.011(0.002)	0.000		0.003(0.001)	0.000		0.005(0.001)	0.000	
**Calcium/vitamin D user (yes/no)**	-	-		-	-		-	-	
**Current smoker (yes/no)**	2.261(4.197)	0.590		-	-		0.359(1.898)	0.850	
**Current drinker (yes/no)**	-	-		-	-		-	-	

*B* = Adjusted unstandardized regression coefficient. SE = standard error for the adjusted unstandardized regression coefficient. R^2^ = Coefficient of multiple determination

^a^ Model adjusted for age, ethnicity and school residency

^b^ Model adjusted for age and school residency

^c^ Model adjusted for age and school residency

## Discussion

The calcaneus QUS technique used in this present study demonstrated a strong correlation between QUS indices and mechanical forces variables (body anthropometric, composition and physical activity). Since calcaneus is a weight-bearing bone, it has been recommended as an appropriate skeletal site for assessing the impact of integrated physical activity on bone. Furthermore, calcaneus bears most of the body weight and faces maximal ground-reaction forces (GRF) as the heel strikes during locomotion [36]. Moreover, QUS measurement of the calcaneus is the most common and the only validated skeletal site used for QUS assessment [37]. Calcaneus is the preferred site because it is predominantly composed of 95% trabecular bone which has a high metabolic turnover rate similar to the spine and is more vulnerable to age, disease and therapy-induced bone alterations than cortical bone [38]. Ultrasound measurement of the calcaneus can accurately measure bone mineral status, thus abating the influence of soft tissue thickness variations [39].

The current study was consistent with those of earlier studies using calcaneal QUS. In a homogenous population of adolescent Caucasian females (aged 14–18 years old), Robinson and colleagues found a significant association between moderate-to-high impact physical activity and calcaneal SI [40]. Moreover, in a large sample of Japanese children and adolescents (aged 12–15 years old), current exercise habit was significantly associated with calcaneal osteo-sono assessment index (OSI) in both genders [41]. Cumulative evidence suggested that exercise might significantly improve QUS values across the age spectrum [42]. Increased physical activity during adolescence has been acknowledged to influence high bone mineral acquisition, an important determinant of good bone status in later life [43]. Bone is most sensitive to the osteogenic effects of physical activity during growth because the bone matrix is in a dynamic state that enables the bone tissue to restructure and grow to withstand mechanical load [44].

It was originally anticipated that the anthropometric variable, which indicates skeletal development, to correlate with QUS measurements of the calcaneus. Previous children and adolescent QUS studies have found a significant association between BMI or weight and calcaneal QUS in both genders [41, 45, 46]. Our result on the other hand demonstrated that BMI was significantly associated with SI and SOS in adolescent females, whereas no significant result was established for BMI in adolescent males. However, our result was in concordance with the previous study among Spanish children (age 4–16 year old), which demonstrated a significant association between weight and phalanges BUA in females, but not in males [47]. A similar finding has also been observed among Caucasian adolescent females [40]. Apart from this discordant result concerning the gender which was possibly due to the differences in hormonal status, the idea behind such a correlation is based on the theory that mechanical stress from body weight can act on the periosteum, which further stimulates bone formation and later triggers expansion of the bone’s periosteal circumference [48].

Exploring the independent association of fat and lean mass in this age-group is important as prior evidence has yielded conflicting results of the positive [49, 50] and negative [51, 52] effects of fat mass on bone mass. Our data demonstrated that fat mass has a negative association with SI in female adolescents, whereas no significant association was found in male adolescents. This result suggested a tendency for adolescent females with extra fat mass to have a negative SI and consequently an increase of the risk of fractures. Our findings were in agreement with those reported in previous studies [47, 53]. This might possibly be linked to the stromal cell origin shared by osteoblasts and adipocytes, whereby in obese person, cell differentiation may favour adipocyte formation—thus inhibit osteoblast differentiation and consequently reduce bone formation [54].

It is noteworthy to mention that adolescent females and males with higher lean mass have significantly higher BUA variance. Our results lend support to an earlier study conducted among Lebanese children (aged 11–18 years old), which showed a positive association of lean mass with phalangeal bone transmission time (BTT) and AD-SOS, in females and males respectively [53]. As others have previously highlighted, lean mass is more beneficial to bone compared to fat mass [48, 55]. The importance of lean mass is based on the mechanostat theory, whereby bone is seen to be more adapted to greater force exerted by the increase in dynamic loads from muscle force, rather than the static loads from fat mass [21, 56].

In agreement with previous studies, the variance for SOS, BUA and SI explained by the chosen variables in the current study were greater for females. These findings forecast a better outcome of intervention for adolescent females to achieve healthy bones, seeing that women (age 50 years or more) are more prone to experience osteoporotic fractures than men [57]. Increasing physical activity, reducing fat mass and increasing lean mass while aiming for a healthy BMI will confer benefits in terms of stronger and healthy bones. A structured programme or intervention based on physical activity that aims to increase lean mass and reduce fat mass will be of the utmost benefit to adolescent females.

Our study was unsuccessful in proving significant association between smoking status and bone status among adolescent males via the multiple regression models. Furthermore, the influence of smoking status in female adolescents and alcohol drinking status could not be tested in the multivariable regression analysis because of low respondent rate and short period of exposure. Despite this, there is prospective evidence that proved early initiation of alcohol drinking and smoking as a predictor of lower bone mineral density in late adolescence [35]. Since smoking has been linked with low bone density and other unhealthy lifestyle such as alcohol use and sedentary behaviors, alcohol consumption at a young age would have a similar adverse effect on bone health as well as an increased risk for bone loss and susceptibility to fracture later in life [58]. Moreover, the present study revealed no significant correlation between intake of calcium, or combination with vitamin D supplement with any QUS parameters in the bivariate regression analyses. The minimal effect of calcium supplementation on children and adolescent bone density may explain this current finding [59].

We acknowledged that there were limitations to our study which prevented causality from being inferred due to the nature of its cross-sectional design. To mitigate this, we need to conduct a prospective longitudinal study in this group to confirm the causal relationship. We are aware that the menarche age in female adolescents is not a perfect substitute (i.e. proxy) variable for pubertal development. This substitute variable was used as we were unable to evaluate pubertal stage in our population as the study was conducted outside of hospital setting where proper physical examination (assessment of secondary characteristics) could be done by paediatrician or physician. Saying that, this idealized setting is not feasible in practice for population studies due to the large sample size [35]. Another limitation was that the data were collected via self-administered questionnaires that may be prone to recall biases.

Despite these limitations, our study serves an important source of information for future paediatric calcaneal QUS studies. This study also lends support to the use of QUS for epidemiological studies in a multi-racial society like Malaysia and other Southeast Asian countries with similar demographic profiles. It should be noted that 55% of worldwide osteoporotic fractures risk (as determined by country-specific FRAX models) were from Asia and this prevalence is set to escalate in the future with the increase of life expectancy, population of elderly people and sedentary lifestyle [60]. Despite this, more than half of adolescents (57.3%) in Malaysia were physically inactive [61], indicating that there is an alarming ‘inactivity epidemic’ currently taking place [62]. Therefore, this study calls for immediate and effective evidence-based intervention approaches for adolescents to be more physically active in order to gain the health benefits before it is too late.

## Conclusions

QUS of the calcaneus is positively influenced by physical activity, body mass index and lean mass, while negatively influenced by fat mass. Promoting health benefits from physical activity by setting the target of healthy body mass index (along with high lean mass and low fat mass) could influence positive bone status and consequently improve peak bone mass, which is a potent protective determinant against osteoporosis in adulthood. The results of this study might potentially assist researchers to develop effective intervention programmes aiming at reinforcement of a healthy and physically active lifestyle. This might bestow a positive impact regarding the augmentation of adolescents’ bone health and subsequently reducing the morbidity and economic burden of osteoporosis later in life.
